# Impact of Autologous and Allogeneic Stem Cell Transplantation in Peripheral T-Cell Lymphomas

**DOI:** 10.1155/2010/320624

**Published:** 2010-12-21

**Authors:** Peter Reimer

**Affiliations:** Klinik für Hämatologie, Internistische Onkologie und Stammzelltransplantation, Kliniken Essen Süd, Evangelisches Krankenhaus Essen-Werden gGmbH, Pattbergstra*β*e 1-3, 45239 Essen, Germany

## Abstract

Peripheral T/NK-cell lymphomas (PTCLs) are rare malignancies characterized by poor prognosis. So far, no standard therapy has been established, due to the lack of randomised studies. High-dose therapy and autologous stem cell transplantation (HDT-autoSCT) have shown good feasibility with low toxicity in retrospective studies. In relapsing and refractory PTCL several comparison analyses suggest similar efficacy for PTCL when compared with aggressive B-cell lymphoma. In the upfront setting, prospective data show promising results with a long-lasting overall survival in a relevant subset of patients. Achieving a complete remission at transplantation seems to be the most important prognostic factor. Allogeneic stem cell transplantation (alloSCT) has been investigated only as salvage treatment. Especially when using reduced intensity conditioning regimen, eligible patients seem to benefit from this approach. To define the role for upfront stem cell transplantation a randomised trial by the German High-Grade Non-Hodgkin Lymphoma Study Group comparing HDT-autoSCT and alloSCT will be initiated this year.

## 1. Introduction

Peripheral T-cell lymphomas (PTCLs) represent approximately 10%–15% of all non-Hodgkin's lymphomas (NHLs) in Western countries [1–3]. Although the clinical appearance and the manifestation sites vary widely between the different subgroups, most PTCL share some characteristics. Most patients are of older age (median age >60 years) and usually present with advanced stage disease [4, 5]. PTCLs in general show an aggressive course and most studies detect the T-cell phenotype as an independent negative prognostic factor [6–9]. Both, the international prognostic index (IPI) and the prognostic index for T-cell lymphomas (PITs) that also include the bone marrow involvement, have shown prognostic value in PTCL and determine the outcome of patients with nodal PTCL [10–13]. In addition, in retrospective studies further parameters like the expression of Ki-67, the level of *β*_2_-microglobulin, and the detection of the Epstein-Barr virus (EBV) have been found to have some prognostic relevance in PTCL [14–16].

The prognosis of PTCL is poor with the exception of the ALK (anaplastic lymphoma kinase) expressing anaplastic large cell lymphoma (ALCL) with a more favourable outcome after conventional chemotherapy and the primary cutaneous T-cell lymphomas (CTCL) that usually show an indolent clinical course [17, 18]. In contrast, for the remaining PTCL the outcome following anthracycline-based chemotherapy is worse compared to aggressive B-cell lymphomas even regarding the pre-rituximab era with a median overall survival (OS) of 9 to 42 months [19–21].

So far, no accepted standard treatment could be defined for PTCL. This mainly results from a lack of PTCL-restricted randomised trials and the heterogeneity of most published series. Although the CHOP (cyclophosphamide, doxorubicin, vincristine, and prednisone) and CHOP-like regimen are widely used first-line, these protocols have never been established prospectively in PTCL and are rather adopted from treatment strategies for aggressive B-cell lymphomas. Even the role of anthracyclines in the first-line treatment of PTCL is controversial since a large retrospective international survey did not reveal a significant impact on OS [5].

To improve the treatment results in PTCL more aggressive strategies such as high-dose therapy with autologous stem cell transplantation (HDT-autoSCT) and allogeneic stem cell transplantation (alloSCT) seem attractive strategies in PTCL. In this paper the data on stem cell transplantation for PTCL will be discussed.

## 2. Autologous Stem Cell Transplantation

### 2.1. Second-Line Therapy

HDT-autoSCT has become the standard of care in relapsing and primary refractory high-grade B-cell lymphomas. In PTCL, prospective randomized studies on salvage HDT-autoSCT are lacking. To date, at least 16 retrospective studies, each including more than 15 patients have addressed this issue and are listed in Table 1 [22–37].

The cited studies were heterogeneous in terms of histological subgroups, patient characteristics, prognostic factors, myeloablative regimen, and duration of follow-up. In addition, some series included patients receiving upfront autoSCT and did not provide separate analyses for the patients treated in second line. Taken together, this strategy is feasible and safe with a low morbidity and mortality rate. The OS in these series ranged from 35% at 2 years to 70% at 5 years, respectively, and the disease-free survival (DFS) or event-free survival (EFS) from 28% at 2 years to 56% at 5 years, respectively. Although the earlier reports tend to show somewhat better results than the series published recently, when subgroup or matched control analyses were performed, the OS results for PTCL were equivalent to the long-term outcome in patients with aggressive B-cell lymphomas [22, 26, 32]. So far, it is unclear whether histology impacts the outcome of PTCL after salvage autoSCT. In some series ALCL showed a favorable outcome compared to other pathological subtypes [25, 26, 30, 31]. However, the ALK status was not determined in all series and furthermore, Zamkoff et al. could not demonstrate a long-term DFS for recurrent (ALK-negative) ALCL following HDT-autoSCT [29]. The encouraging results for patients with ALCL by Fanin et al., (5-year OS and PFS of 70% and 56%, resp.) were probably biased by age and inclusion of patients in first complete remission (CR), who showed a significantly better outcome in a subgroup analysis [23].

The disease status at the time of transplantation often correlates with the outcome after salvage HDT-autoSCT. In fact, several authors found a better long-term survival in patients transplanted in CR than in patients with other disease status at transplantation [23, 27, 32, 33, 35–37]. Other authors could not confirm this finding in their survey [24, 25, 31]. However, since all data in this setting are generated retrospectively, the value of this observation needs further observation.

In summary, second-line HDT-autoSCT in PTCL is feasible and seems an effective approach for a considerable subgroup of patients.

### 2.2. First-Line Therapy

Some retrospective studies on upfront HDT-autoSCT have been published and are summarized in Table 2(a) [38–43]. Like in the salvage setting, a comparison of the cited series is hampered by their variety. Some series reported mainly on patients with a low or intermediate low IPI, whereas others predominantly included patients with an unfavorable prognostic index. In addition, most studies contained patients receiving HDT-autoSCT in second line, but not all of them show a subgroup analysis for the upfront setting. The OS in these retrospective studies ranged from 53% at 3 years to 62–68% at 5 years. Interestingly, the DFS/EFS did not appear to be much lower than the OS in most cohorts that might indicate a substantial curative potential for this approach in previously untreated PTCL. The EBMT (European Group for Blood and Marrow Transplantation) published the largest study in this setting. Kyriakou et al reported data on 146 patients with angioimmunoblastic T-cell lymphoma (AITL) showing an actuarial OS of 67% at 2 years and 59% at 4 years, respectively, after a median observation of 31 months. About two thirds of the patients were transplanted in first CR or PR. Interestingly, patients who received a TBI- (total body irradiation-) based conditioning regimen had a significantly lower relapse rate in this study [41].

In these retrospective studies chemotherapy-sensitive disease was the major factor predicting OS and PFS [39–41, 43]. Patients transplanted in CR or PR showed a superior long-term outcome compared to patients with chemotherapy-refractory disease. Other parameters (e.g., age, PIT, IPI) could not consistently be detected as being of prognostic value.

Although mainly showing promising results, the cited retrospective studies are limited by focussing on patients, only, who actually proceed to transplantation leading to superior results due to patient selection.

Prospective randomized PTCL-restricted studies assessing the value of upfront high-dose therapy in PTCL are lacking. Two French trials by the GELA (Groupe d'Etude des Lymphomes de l'Adulte) published data on autoSCT as frontline strategy in poor-risk, aggressive NHL, including PTCL [57–59]. In the LNH87-2 study, patients were treated with either consolidative sequential chemotherapy or HDT-autoSCT [57, 58]. The LNH93-3 trial compared a high-dose arm with shortened first-line myeloablative chemotherapy with a sequential consolidation chemotherapy arm. In the intent-to-treat analysis, none of these studies demonstrated a significant benefit for the high-dose arm [59]. In addition, a pooled data matched control analysis failed to show a significant advantage for upfront HDT-autoSCT [44, 60]. However, the limited number of patients in the high-dose group and the restriction to high-risk patients, only, do not allow to definitely clarify the impact of first-line HDT-autoSCT in PTCL from these data. In another subgroup analysis, Nickelsen et al. reported results of 33 patients with PTCL from a single-arm study by the German High-Grade Non-Hodgkin Lymphoma Study Group [45]. Patients with high-risk aggressive lymphomas were treated with dose-escalated CHOP plus etoposide necessitating repeated autoSCT. Compared to B-cell NHL, PTCL showed a significantly worse OS and EFS at 3 years in an intent-to-treat analysis.

So far, five larger prospective PTCL-restricted trials have published data on 372 patients with frontline HDT-autoSCT [46–50]. Compared to the cited retrospective studies, these prospective series are much more homogeneous. The median age ranged between 43 and 57 years, PTCL unspecified; AITL and ALCL accounted for 77 to 100% of all histological subtypes; the age-adjusted IPI was high or intermediate high in 46 to 72%; the most commonly used myeloablative regimen was the BEAM protocol (*n* = 228), and the disease status at transplantation was CR or PR in 59 to 76%. Only one study included ALK-positive ALCL [46]. In these trials the OS ranged from 48 to 73% at 3 years to 34% at 12 years. The DFS/EFS or the progression-free survival (PFS) was between 36 to 53% at 3 years and 30% at 12 years. One consistently found problem of upfront HDT-autoSCT is early progressive disease leading to about one third of patients in intent-to-treat analyses, who finally fail to achieve transplantation. Mercadal et al. reported a still significant lower transplantation rate of 41%. Of note, in this trial poor stem cell mobilization was the second most frequent cause of failing HDT-autoSCT [48].

With regard to these prospective data, again the remission status at the time of transplantation was a significantly prognostic factor in most studies that provided this analysis [46–48]. In addition, the IPI and PIT also show prognostic value in some [48, 49] but not in all series [47]. Other parameters, for example, histological subtype, age, sex, stage have not been concordantly been found to impact the outcome. The prospective series on upfront autoSCT are listed in Table 2(b).

## 3. Allogeneic Stem Cell Transplantation

In contrast to the cytotoxic effect of HDT-autoSCT, allogeneic SCT (alloSCT) could add a graft-versus-lymphoma (GVL) effect to the myeloablative or reduced intensity conditioning (RIC) regimen, potentially improving the therapeutic outcome. However, the experience with alloSCT for PTCL is limited. To date, no relevant data for the upfront setting are available. Besides some case reports, five retrospective series with at least 10 patients have been reported in patients with relapsing and refractory PTCL (Table 3(a)) [40, 51–54].

The largest series was published by the Société Française de Greffe de Moëlle et de Thérapie Cellulaire. In 77 pretreated patients who mainly had a myeloablative conditioning regimen the 5-year OS and PFS were 57% and 53%, respectively, after a median followup of 43 months. The treatment-related mortality (TRM) was 33% at 5 years. In a multivariate analysis, chemotherapy-resistant disease at transplantation and grade 3/4 acute graft-versus-host disease (GVHD) were the strongest adverse prognostic factors for OS. The TRM was similar in both conditioning groups [53]. Most studies could reveal a GVL effect [52–54]. However, the TRM/NRM (nonrelapse mortality) had a relevant impact on outcome and was increasing over time up to 69% at 3 years in the series by Hamadani et al. [52]. The OS ranged from 40% at 2 years to 57% at 5 years. 

Two prospective studies have been published so far (Table 3(b)) [55, 56]. In the Italian phase II trial by Corradini et al. 17 patients underwent RIC and alloSCT as salvage therapy [55]. Eight out of 17 patients had failed front-line HDT-autoSCT. After a median followup of 28 months, 14 of 17 patients were alive. The estimated 3-year OS and PFS rates were 81% and 64%, respectively. The TRM was impressively low with only 6%. Notably, donor lymphocyte infusions given at the time of progression resulted in a disease response in two out of four patients, indicating evidence of a GVL effect. In the German study by Wulf et al. 10 patients were treated with chemotherapy combined with the humanized antiCD52 monoclonal antibody, alemtuzumab, followed by RIC and alloSCT. Two patients had prior HDT-autoSCT. The OS was 70% with six patients in CR after a median followup of 7 months [56].

## 4. Summary

Due to their generally poor prognosis after conventional chemotherapy more effective treatment strategies in PTCL are urgently needed. Although randomised trials are lacking, HDT-autoSCT can be regarded as feasible and safe in PTCL. In the salvage setting, several subgroup analysis and comparisons show similar results compared to diffuse large-cell B-cell lymphoma. This finding could recently be confirmed by Sohn et al. [61]. Therefore, taken together the existing data, HDT-autoSCT seems a reasonable approach in relapsing and refractory PTCL particularly in those with chemotherapy-sensitive disease.

The value of upfront HDT-autoSCT remains to be definitely established. A recently published retrospective comparison did not find a significant benefit of this strategy compared to conventional treatment [62]. However, in this study the high-dose group was heterogeneous ranging from high-dose CHOP to alloSCT. In contrast, other retrospective studies revealed a significant better outcome when HDT-autoSCT was compared to chemotherapy, alone [5, 36]. Taken together, the prospective data mainly show promising results, especially for patients achieving a good remission status prior to transplantation, that has been reported as independent prognostic factor in most series. Therefore, these patients should mainly be regarded as candidates for upfront autoSCT. Since one major obstacle of this approach is early progressive disease, novel treatment concepts incorporating new agents and/or dose-dense regimen should be further investigated to improve remission status prior to transplantation. In a subanalysis of several trials by the DSHNHL (German High-Grade Non-Hodgkin Lymphoma Study Group), the addition of etoposide to the CHOP-protocol improved the outcome in younger patients with PTCL [63]. Furthermore, alemtuzumab has shown efficacy especially in untreated PTCL [64–67]. However, this agent can cause significant infectious and hematologic toxicities that have led to early closure of some trials [65–67]. Recently, EBV-associated B-cell lymphomas have been reported complicating alemtuzumab therapy especially when given in higher dosages [68, 69]. To better define the role of chemoimmunotherapy in the concept of HDT-autoSCT, the Nordic Lymphoma Group is conducting a multicenter randomized trial using dose-dense chemotherapy induction with or without alemtuzumab.

Allogeneic stem cell transplantation could offer a curative option in younger patients. However, the experience with this approach is sparse and limited to relapsed and refractory PTCL. In the prospective series nonmyeloablative conditioning protocols were used with very encouraging results especially in the Italian study. Furthermore, a GVL effect could be demonstrated. Taken together, the current data support the concept of alloSCT in eligible patients with relapsing chemosensitive PTCL, especially after failing prior HDT-autoSCT.

To further investigate the role of stem cell transplantation in previously untreated PTCL, this year the DSHNHL in cooperation with other groups will initiate a prospective randomised multicenter trial comparing upfront autoSCT versus alloSCT following dose-dense induction chemotherapy. 

## Figures and Tables

**Table 1:** Studies on high-dose therapy and autologous stem cell transplantation in PTCL as second-line therapy.

Retrospective data											
Author	Year	*n*	Age	Histologies (WHO)	IPI	High-dose regimen	Status at Tx	DFS/EFS/ PFS/RFS	OS	Followup (months)	Comment
Vose et al. [22]	1990	17	33	No data*	No data	Diverse	CR 42% PR 26%	28% (2 y)	35% (2 y)	28	

Fanin et al. [23]	1999	64	25	ALCL 100%	aaIPI 0/1 48% 2/3 22% Unknown 30%	Diverse	CR 47%	56% (5 y)	70% (5 y)	43	34/64 transplanted 2.line

Rodríguez et al. [24]	2001	29	43	No data*	0/1 31% 2 41% 3 21% 4/5 7%	Diverse	CR 38% PR 48%	32% (3 y)	39% (3 y)	43	

Blystad et al. [25]	2001	40	42	PTCLu 50% ALCL 35% Other 15%	aaIPI 0/1 42% 2/3 45% Unknown 13%	Diverse	CR 70% PR 30%	56% (3 y)	58% (3 y)	25	23/40 transplanted 2.line

Song et al. [26]	2002	36	46	PTCLu 56% ALCL 25% NK/T 11% Other 8%	No data	Mel/Eto	CR 42% PR 50%	37% (3 y)	48% (3 y)	42	

Rodríguez et al. [27]	2003	115	41	PTCLu 63% ALCL 22% NK/T 15%	aaIP 0/1 40% 2/3 60%	Diverse	CR 56% PR 38%	60% (5 y) 49% (5 y)**	56% (5 y) 45% (5 y)**	37	78/115 transplanted 2.line

Schetelig et al. [28]	2003	29	51	AITL 100%	aaIPI 0/1 21% 2/3 79%	Diverse	No data	37% (5 y) 39% (5 y)**	60% (5 y) 44% (5 y)**	60	15/29 transplanted 2.line

Zamkoff et al. [29]	2004	16	52	ALK^−^ALCL 100%	aaIPI 0/1 67% 2/3 33%	Diverse	CR 60% PR 40%	12 w (median)	72 w (median)	No data	

Jantunen et al. [30]	2004	37	46	PTCLu 38% ALCL 38% EATL 14% Other 11%	0/1 46% 2 22% 3 19% 4/5 14% Unknown 3%	BEAC/BEAM	CR/PR 87%	44% (5 y) 28% (5 y)**	54% (5 y) 45% (5 y)**	24	19/37 transplanted 2.line TRM 16%

Jagasia et al. [31]	2004	28	39	ALCL 57% PTCLu 21% AITL 11% NK/T 11%	aaIPI 0/1 29% 2/3 50% Unknown 21%	Cy/Eto/TBI or CBV	CR 39% PR 46%	50% (3 y)	69% (3 y)	44	7/28 underwent alloSCT

Kewalramani et al. [32]	2006	24	48	PTCLu 58% ALCL 17% AITL 17% Other 8%	aaIPI 0/1 46% 2/3 54%	Diverse	CR 63% PR 37%	24% (5 y)	33% (5 y)	72	

Kim et al. [33]	2007	40	44	PTCLu 50% NK/T 25% ALCL 13% Other 13%	aaIPI 0/1 45% 2/3 53% Unknown 3%	Diverse	CR 28% PR 52%	No data	11.5 m (median)	16	29/40 transplanted 2.line

Smith et al. [34]	2007	32	44	ALCL 66% PTCLu 34%	aaIPI (at Tx) 0/1 72% 2/3 28%	BEC	No data	18% (5 y)	34% (5 y)	30	26/32 transplanted 2.line

Chen et al. [35]	2008	53	45	ALCL 34% PTCLu 30% AITL 17% Other 19%	No data	Diverse	CR/PR 89%	25% (5 y) 9% (5 y)**	48% (5 y) 37% (5 y)**	60	38/53 transplanted 2.line

Lee et al. [36]	2008	47	42	NK/T 100%	aaIPI 0/1 82% 2/3 18%	CVB/BEAM/ MCEC (72%)	CR 58%	No data	No data	117	33/47 transplanted 2.line

Yang et al. [37]	2009	64	44	PTCLu 100%	aaIPI 0/1 56% 2/3 44%	BEAM/CVB (70%)	CR 33% PR 58%	44% (3 y) 33% (3 y)**	53% (3 y) 46% (3 y)**	30	36/64 transplanted 2.line

Studies including both patients receiving HDT-autoSCT 1.line and 2.line are listed in the table representing the predominant group

*classified according to the Working Formulation

**for the subgroup of patients transplanted 2.line.

**Table:** (a) Retrospective data

Author	Year	*n*	Age	Histologies (WHO)	IPI	High-dose regimen	Status at Tx	DFS/EFS/ PFS/RFS	OS	Followup (months)	Comment
Rodríguez et al. [38]	2007	19	46	AITL 100%	aaIPI 0/1 37% 2/3 63%	BEAM/BEAC (79%)	CR1 42% PR1 26%	55% (3 y)	60% (3 y)	25	15/19 transplanted 1.line

Rodríguez et al. [39]	2007	74	46	PTCLu 50% ALCL 31% AITL 11% Other 7%	aaIPI 0/1 35% 2/3 65%	BEAM/BEAC (91%)	No data	63% (5 y)	68% (5 y)	67	

Feyler et al. [40]	2007	64	45	PTCLu 47% ALCL 31% AITL 8% Other 14%	0/1 34% 2 11% 3 11% 4/5 2% Unknown 42%	Diverse	CR1 48% PR1 23%	50% (3 y)	53% (3 y)	48	Incl. CTCL, T-cell leukemia/lymphoma 46/64 transplanted 1.line

Kyriakou et al. [41]	2008	146	53	AITL 100%	No data	BEAM (74%)	CR1 33% PR 36%	49% (4 y)	59% (4 y)	31	101/146 transplanted 1.line

Prochazka et al. [42]	2009	18	59	PTCLu 56% ALCL 39% AITL 6%	0/1 28% 2 28% ≥3 44%	BEAM	No data	52% (2 y)	71% (2 y)	26	

Numata et al. [43]	2010	39	53	PTCLu 31% AITL 28% ALCL 23% NK/T 18%	aaIPI 0/1 33% 2/3 59% Unknown 8%	MCEC (*n* = 32) TBI-based (*n* = 7)	CR1 69%	61% (5 y)	62% (5 y)	78	23/39 transplanted 1.line

Studies including both patients receiving HDT-autoSCT 1.line and 2.line are listed in the table representing the predominant group.

**Table:** (b) Prospective data

Author	Year	*n*	Age	Histologies (WHO)	IPI	High-dose regimen	Status at Tx	Tx rate	DFS/EFS/ PFS	OS	Followup (months)	Comment
Mounier et al. [44]	2004	28	36	PTCLu 56% Precursor 44%	0/1 25% ≥2 75%	BEAM/CBV	CR 100%	No data	44% (5 y)	54% (5 y)	78′	Incl. precursor T-cell lymphoma

Nickelsen et al. [45]	2009	33	48	ALK^−^ALCL 39% PTCLu 33% AITL 12% Other 16%	aaIPI 0/1 9% 2/3 91%	MegaCHOEP	CR 49% PR 6% after therapy	67%	26% (3 y)	45% (3 y)	53	Sequential HDT-autoSCT Subgroup analysis

Corradini et al. [46]	2006	62	43	PTCLu 45% ALK^+^ ALCL 30% AITL 16% Other 9%	0/1 19% ≥2 71%	Mito/Mel or BEAM	CR 56% PR 16%	71%	30% (12 y)	34% (12 y)	76	Incl. ALK^+^ALCL

Rodríguez et al. [47]	2007	26	44	PTCLu 42% ALK^+^ALCL 31% AITL 27%	aaIPI 0/1 28% 2/3 72%	BEAM	CR 65% PR 8%	73%	53% (3 y)	73% (3 y)	35'	No ALK^+^ALCL 13/26 transplanted 1.line

Mercadal et al. [48]	2008	41	47	PTCLu 49% AITL 29% HSTL 5% NK/T 5% Other 12%	0/1 22% 2 32% 3 22% 4/5 24%	BEAM/BEAC	CR 49% PR 10%	41%	30% (4 y)	39% (4 y)	38	No ALK^+^ALCL

Reimer et al. [49]	2009	83	47	PTCLu 39% AITL 33% ALK^−^ALCL 16% Other 12%	aaIPI 0/1 49% 2/3 51%	Cy/TBI	CR 47% PR 24%	66%	36% (3 y)	48% (3 y)	33	No ALK^+^ALCL

D'Amore et al. [50]	2009	160	57	PTCLu 39% AITL 19% ALK^−^ALCL 19% HSTL 13% Other 10%	0/1 18% ≥2 72%	BEAM	No data	71%	49% (3 y)	57% (3 y)	45	No ALK^+^ALCL

**Table:** (a) Retrospective data

Author	Year	*n*	Age	Histologies	IPI	Previous autoSCT	Regimen (MA versus RIC)	Status at Tx	DFS/EFS/ PFS	OS	TRM/NRM	Followup (months)	GVHD
Feyler et al. [40]	2007	18	28	PTCLu 50% T-cell leuk. 28% ALCL 17% CTCL 6%	0/1 33% ≥2 66%	11%	100% MA	No data	33% (3 y)	39% (3 y)	38%*	57	Acute GVHD °3/4: 28% Ext. chronic GVHD: 6%

Murashige et al. [51]	2005	28	38	NK/T 79% Blastic NK 11% NK-leukemia 11%	No data	32%	82% versus 18%	CR 57%	34% (2 y)	40% (2 y)	29%	34	Acute GVHD °3/4: 29% Ext. chronic GVHD: 11%

Hamadani et al. [52]	2008	14	43	PTCLu 36% AITL 28% ALCL 14% NK/T 14% Other 7%	aaIPI 0/1 57% 2/3 42%	14%	57% versus 43%	CR 21% PR 35%	31% (3 y)	35% (3 y)	28%*	34	Acute GVHD °3/4: 21% Chronic GVHD: 50%

Le Gouill et al. [53]	2008	77	36	PTCLu 35% ALCL 35% AITL 14% Other 16%	0/1 61% ≥2 32% Unknown 6%	25%	74% versus 26%	CR 40% PR 30%	53% (5 y)	57% (5 y)	21%*	43	Acute GVHD °3/4: 21%

Kyriakou et al. [54]	2009	45	48	AITL 100%	No data	33%	56% versus 45%	CR 27% PR 22%	53% (3 y)	64% (3 y)	18%*	29	Acute GVHD °3/4: 11% Ext. chronic GVHD: 24%

**Table:** (b) Prospective data

Author	Year	*n*	Age	Histologies	IPI	Previous autoSCT	Regimen (MA versus RIC)	Status at Tx	DFS/EFS/ PFS	OS	TRM/NRM	Followup (months)	GVHD
Corradini et al. [55]	2004	17	41	PTCLu 53% ALCL 24% AITL 24%	aaIPI 0/1 24% 2/3 76%	47%	100% RIC	CR 12% PR 71%	64% (3 y)	81% (3 y)	6%	28	Acute GVHD °3/4: 12% Ext. chronic GVHD: 6%

Wulf et al. [56]	2005	10	45	PTCLu 40% ALCL 30% AITL 20% T-PLL 10%	No data	20%	100% RIC	CR 10% PR 50%	60% (7 m)	70% (7 m)	30%	7	Acute GVHD °3/4: 10% Ext. chronic GVHD: 50%

*at day 100.

## Abbreviations

AaIPI: Age-adjusted IPI
ALCL: Anaplastic large cell lymphoma
ALK: Anaplastic lymphoma kinase
AlloSCT: Allogeneic stem cell transplantation
AITL: Angioimmunoblastic T-cell lymphoma
BEAC: BCNU, etoposide, cytarabin, cyclophosphamide
BEAM: BCNU, etoposide, cytarabin, melphalan
BCNU: Carmustine
BEC: Busulfan, etoposide, cyclophosphamide
CR: Complete remission
CVB: BCNU, etoposide, cyclophosphamide
DSF: Disease-free survival
EATL: Enteropathy-associated T-cell lymphoma
EFS: Event-free survival
Eto: Etoposide
IPI: International prognostic index
M: Months
Mel: Melphalan
NK/T: Natural killer-cell/T-cell leukemia/lymphoma
OS: Overall survival
PFS: Progression-free survival
PR: Partial remission
PTCLu: Peripheral T-cell lymphoma, unspecified
Thio: Thiotepa
TRM: Treatment-related mortality
Tx: Transplantation
W: Weeks
Y: Year
CHOEP: Cyclophosphamide, vincristine, doxorubicin, etoposide, prednisone
CTCL: Cutaneous T-cell lymphoma
CVB/CBV: BCNU, etoposide, cyclophosphamide
Cy: Cyclophosphamide
HDT-autoSCT: High-dose therapy with autologous stem cell transplantation
HSTL: Hepatosplenic T-cell lymphoma
MCEC: Ranimustine, cyclophosphamide, etoposide, carboplatin
Mito: Mitoxantrone
TBI: Total body irradiation
AutoSCT: Autologous stem cell transplantation
Ext: Extensive
GVHD: Graft-versus-host disease
Leuk: Leukemia
MA: Myeloablative conditioning
NK: Natural killer cell
NRM: Nonrelapse mortality
RIC: Reduced intensity conditioning
T-PLL: T-cell prolymphocytic leukaemia.
